# At-line Prediction of Gelatinized Starch and Fiber Fractions in Extruded Dry Dog Food Using Different Near-Infrared Spectroscopy Technologies

**DOI:** 10.3390/ani10050862

**Published:** 2020-05-16

**Authors:** Arianna Goi, Carmen L. Manuelian, Federico Righi, Massimo De Marchi

**Affiliations:** 1 Department of Agronomy, Food, Natural resources, Animals and Environment, University of Padova, Viale dell’Università 16, 35020 Legnaro (PD), Italy; arianna.goi@unipd.it (A.G.); massimo.demarchi@unipd.it (M.D.M.); 2 Department of Veterinary Science, University of Parma, Via del Taglio 10, 43126 Parma, Italy; federico.righi@unipr.it

**Keywords:** fibrous fractions, NIT, non-fibrous carbohydrates, Vis-NIR, pet food, starch gelatinization

## Abstract

**Simple Summary:**

Starch is a non-fibrous carbohydrate that represents an important percentage of pet food composition. The degree of its gelatinization, due to the cooking process, can be a useful indicator of starch digestibility in the diet. Moreover, fiber fractions are important for animals’ health and nutritional status, so pet food industry is interested in the development of an easy and cost-effective method to measure these parameters. Results of this study revealed the applicability of visible/near-infrared spectroscopy to predict total and gelatinized starch, neutral detergent fiber, acid detergent fiber, and acid detergent lignin in pet food. On the other hand, near-infrared transmittance technology showed a scarce accuracy. The developed prediction models for total and gelatinized starch and fiber fractions using visible/near-infrared spectroscopy could be applied during the manufacturing process to perform quality controls.

**Abstract:**

This study aimed to assess the feasibility of visible/near-infrared reflectance (Vis-NIR) and near-infrared transmittance (NIT) spectroscopy to predict total and gelatinized starch and fiber fractions in extruded dry dog food. Reference laboratory analyses were performed on 81 samples, and the spectrum of each ground sample was obtained through Vis-NIR and NIT spectrometers. Prediction equations for each instrument were developed by modified partial least squares regressions and validated by cross- (CrV) and external validation (ExV) procedures. All studied traits were better predicted by Vis-NIR than NIT spectroscopy. With Vis-NIR, excellent prediction models were obtained for total starch (residual predictive deviation; RPD_CrV_ = 6.33; RPD_ExV_ = 4.43), gelatinized starch (RPD_CrV_ = 4.62; RPD_ExV_ = 4.36), neutral detergent fiber (NDF; RPD_CrV_ = 3.93; RPD_ExV_ = 4.31), and acid detergent fiber (ADF; RPD_CrV_ = 5.80; RPD_ExV_ = 5.67). With NIT, RPD_CrV_ ranged from 1.75 (ADF) to 2.61 (acid detergent lignin, ADL) and RPD_ExV_ from 1.71 (ADL) to 2.16 (total starch). In conclusion, results of the present study demonstrated the feasibility of at-line Vis-NIR spectroscopy in predicting total and gelatinized starch, NDF, and ADF, with lower accuracy for ADL, whereas results do not support the applicability of NIT spectroscopy to predict those traits.

## 1. Introduction

Starch is a polymer organized in concentric semi-crystalline or amorphous layers constituted by amylose and amylopectin [1]. In companion animals food, starch represents up to 50% of the product [2]. Starch generally derives from one or few cereal grains added up to 60% of the ingredients [3,4], and it represents an important source of energy since its digestion releases molecules of glucose. On the other hand, fibrous fractions, such as neutral detergent fiber (NDF), acid detergent fiber (ADF), and acid detergent lignin (ADL), are physiologically important because of their influence on the ingesta transit time, gastric emptying, and fecal volume [5].

Most of the uncooked cereal starch presents a low digestibility in dogs [4]. Therefore, heat treatments applied during manufacture play a fundamental role in improving the digestibility of starch in food. Due to the high proportion of starch in extruded and baked dog diets, the digestibility of these typologies of dog foods is highly related to the digestibility of this component [4], which can also potentially affects the apparent total tract macronutrient digestibility, fecal characteristics, and fecal fermentative end-products in dogs [6]. Extrusion is a widespread technology to produce commercial dry dog food because of its economicity [7] and its capacity to mix, cook, sterilize, and texturize food ingredients [2]. Extrusion modifies starch structure, leading to the release of amylose and amylopectin, which increases the solubility and the capacity to absorb water of this polysaccharide, reducing at the same time its viscosity [8,9]. The complex of these modifications is known as “gelatinization” [10,11].

Extrusion reduces apparent nitrogen digestibility, enhances colonic fermentation—as revealed by a decrease in fecal pH associated to the assumption of extruded food [12]—slightly increases dry and organic matter digestibility likely due to the increase in starch digestibility, and increases ether extract digestibility [13]. From a quality control perspective, the industry is interested in the ratio between gelatinized and total starch, which indirectly expresses its availability. No information is available in literature about the optimal value of this parameter, but as the ratio expresses the amount of digestible starch among the overall quantity, a high value is usually preferable. Sometimes, a small percentage of resistant starch is guaranteed to assure butyrate production due to its beneficial effects on the gut health [14]. Thus, some companies are claiming proportions of gelatinized starch between 90% and 95% in their food products.

Due to the importance of both fibrous fractions and non-fibrous carbohydrates for the animals’ health and nutritional status, a further interest of the pet food industry is the development of an on-line or at-line rapid and cost-effective method to measure these parameters in order to monitor food composition. The common reference analyses to quantify starch, gelatinized starch, and fiber fractions (NDF, ADF, and ADL) are time-consuming, expensive, and need qualified analysts to be performed. On the other hand, near-infrared spectroscopy is already routinely used in most of pet food factories for the determination of gross composition of their products. This method offers a rapid, objective, easy to manage, chemical-free, and non-destructive analysis of several traits simultaneously at a lower cost than the common reference analyses [15,16,17], facilitating quality control.

Infrared spectroscopy capacity to predict food compounds is based on the bonds between hydrogen and elements like C, O, and N (C-H, O-H, and N-H), making total starch, gelatinized starch, and fibrous fractions good candidates to be accurately predicted with this technology. Infrared spectroscopy has already demonstrated its ability to predict proximate composition parameters—such as moisture, protein, and fat—of dog food [18,19,20], starch, and ADL in compound feeds for rabbits [21] and starch, NDF, ADF, and ADL in compound feeds for swine [22]. However, to the best of our knowledge, there is no information regarding the ability of near-infrared spectroscopy to predict starch, gelatinized starch, and fiber fractions in dog food. Therefore, the aim of this study was to assess the feasibility of visible/near-infrared spectroscopy in reflectance mode and near-infrared spectroscopy in transmittance mode to predict total starch, gelatinized starch, and fiber fractions in extruded dry dog food.

## 2. Materials and Methods

### 2.1. Sample Selection

A total of 81 sealed commercial packages of 2, 2.5, and 3 kg of extruded dog food intended for puppies and adults of several breed sizes (Table 1) were collected from March 2018 to April 2019 from a pet food industry in North Italy (Dorado S.r.l.; Monsole di Cona, Venice, Italy). The diversity of the selected products was considered as representative of the availability of extruded dry dog food in the Italian market and provides a high variability that is necessary to develop a prediction model [23,24]. Table 2 shows the chemical composition—dry matter (DM), crude protein (CP), ether extract (EE), and ash—as reported by the manufacturer. Moreover, Tables S1 and S2 report the ingredients and chemical composition, respectively, of each single package included in the study. Once received, packages were stored in absence of light and at room temperature in a dedicated area of the food laboratory of the Department of Agronomy, Food, Natural resources, Animals and Environment of the University of Padova (Legnaro, Italy) until analyses were performed. For the analyses, kibbles (100 g) from each package were grounded in a mill with a 1-mm screen (Retsch Grindomix GM200; Retsch GmbH & Co, Haan, Germany) and divided into two aliquots: one was subjected to chemical analysis, and the other underwent spectrophotometric analysis with two different instruments.

### 2.2. Reference Analysis

Milled samples were analyzed for the total amount of starch using an internal method. Briefly, 500 mg of sample were weighed in a 100-mL PYREX glass tube (SciLabware, Stoke on Trent, the United Kingdom) and added 50 mL of KOH (Carlo Erba, Milano, Italy) 0.5 M; the mixture was quickly mixed with an electric vortex mixer, heated in an shaking water bath oscillating 80 times/min at 60 °C for 60 min, and then cooled to room temperature. Thereafter, glacial acetic acid of a high degree of purity (>95%) (Carlo Erba, Milano, Italy) was added by pipetting to the solution until reaching a pH between 4.6 and 4.8, measured by pH-meter with temperature probe correction. Subsequently, amyloglucosidase solution was produced by adding in a 50-mL PYREX glass flask (Schott Duran, Wertheim, Germany) 22.5 mg of amyloglucosidase standard from *Aspergillus niger* (10115, Sigma-Aldrich, Steinheim, Gemany) with an aliquot of deionized water (Millipore Corporation, Burlington, MA, USA), dissolved in an ultrasonic bath for 10 min, and made up to volume with deionized water. Two milliliters of the amyloglucosidase solution were added to the sample solution and incubated over night at 40 °C for the enzymatic hydrolysis. After cooling, the whole solution was transferred to a 100-mL glass flask (Schott Duran, Wertheim, Germany), diluted with deionized water to capacity, and shaken. Then, an aliquot of 10 mL was transferred into a 10-mL PYREX glass tube (SciLabware, Stoke on Trent, the United Kingdom), centrifuged at 2700× *g* for 10 min at room temperature (20 °C), and the supernatant was filtered through a 0.45-µm filter. Finally, 10 µL of the filtered supernatant was injected in a high-performance liquid chromatography spectra system equipped with an Aminex HPX 87H column (Bio-Rad, Hercules, CA, USA) and aqueous solution of sulfuric acid 0.0025 N as the mobile phase, for the quantification of glucose. The working conditions were: flow rate of 0.6 mL/min and cell internal temperature of 38 °C. A glucose standard solution and a calibration line were made for the quantitative determination of glucose. To prepare the glucose standard solution, 200 mg of D-(+)-glucose anhydrous (G-7528, Sigma-Aldrich, Steinheim, Germany) were weighed in a 50-mL glass flask and were made up to volume with deionized water. Afterwards, for each calibration level, a known aliquot of the glucose standard solution was put in a 25-mL flask making up to volume with sulfuric acid 0.1 N. Starch quantification was performed in duplicate.

Damaged starch quantification was performed using a Megazyme kit (Megazyme Intl. Ireland Ltd., Co., Wicklow, Ireland) following the method 76-31.01 of the American Association of Cereal Chemists (AACC) that consists in an enzyme digestion procedure with purified fulgal α-amylase that only affects damaged starch. The oligosaccharides formed were treated with purified amyloglucosidase to obtain the glucose. Even if quantified damaged starch does not completely correspond to gelatinized starch, considering the extrusion process conditions, we assume that all the damaged starch in the matrix undergo gelatinization, and thus, in this case, the two parameters present comparable values [13,25]. The fiber fractions were also analyzed according to official methods [26]: NDF, method 2002.04, and ADF and ADL, method 973.18.

### 2.3. Near-Infrared Spectroscopy Analysis

Each sample was analyzed using two infrared instruments: a visible/near-infrared spectrophotometer in reflectance mode (Vis-NIR) and a near-infrared spectrophotometer in transmittance mode (NIT). Both instruments worked at room temperature (20 °C) and were located in the same laboratory. The same sample was scanned consecutively with both instruments. Spectra were collected using ISIscan Nova and Mosaic software (FOSS Electric A/S, Hillerød, Denmark).

For the Vis-NIR instrument, 50 g of ground sample were placed in a large FOSS cup (diameter 105 mm, depth 35 mm; FOSS Electric A/S, Hillerød, Denmark) and scanned by NIRS DS2500 (FOSS Electric A/S, Hillerød, Denmark) from 400 to 2500 nm every 0.5 nm. Each spectrum was the average of 32 subspectra collected during the automatic rotation of the FOSS cup and recorded as log(1/reflectance). For the NIT instrument, the same 50-g ground sample was placed in a sample cup (diameter 140 mm, depth 14 mm) and scanned by FoodScan (FOSS Electric A/S, Hillerød, Denmark) from 850 to 1050 nm every 2 nm. Each spectrum was the average of 16 subspectra collected during the automatic rotation of the cup and recorded as log (1/transmittance).

### 2.4. Chemometric Analysis

Chemometric analysis was performed using WinISI 4 software (Infrasoft International, Port Matilda, PA, USA) through modified partial least square regression analysis. The prediction equations for each trait were developed using (i) the complete dataset (*n* = 81) and then tested by leave-one-out cross-validation, and (ii) a subset as the calibration set using 75% of the samples (*n* = 60) and then tested on the remaining 25% of the samples performing an external validation (*n* = 21). To establish the calibration and the validation set, the complete dataset was randomly divided into two subsets with similar mean and standard deviations for each trait. In the calibration procedure, to increase the accuracy in terms of the coefficient of determination and residual predictive deviation, three passes of outliers elimination were applied. The critical *T*-statistic value set for *T*-outliers detection was 2.5, removing the samples whose predicted value deviated more than 2.5 standard errors of cross-validation from the reference value. In order to develop the most accurate calibration models, different combinations of scatter corrections (NONE, no correction; D, detrending; SNV, standard normal variate; SNV + D, standard normal variate and detrending; MSC, multiplicative scatter correction; WMSC, weighted multiplicative scatter correction; and ISC, inverted scatter correction) and mathematical treatments (0,0,1,1; 1,4,4,1; 1,8,8,1; 2,5,5,1; and 2,10,10,1, where the first digit is the number of the derivative, the second is the gap over which the derivative is calculated, the third is the number of data points in the first smoothing, and the fourth is the number of data points in the second smoothing [27]) were applied.

The best calibrations were assessed based on the number of latent factors (LF), the coefficient of determination of calibration (R^2^_C_), cross-validation (R^2^_CrV_), and external validation (R^2^_ExV_); the standard error of calibration (SE_C_), cross-validation (SE_CrV_), and external validation (SE_P_); and the residual predictive deviation of cross-validation (RPD_CrV_) and of external validation (RPD_ExV_), calculated as the ratio of SD to SE_CrV_ or SE_P_, respectively [28]. Residuals of prediction equations obtained performing cross- and external validations were normally distributed, and bias did not differ statistically from zero.

## 3. Results

### 3.1. Chemical Composition of the Samples

Total and gelatinized starch were 34.22 g/100 g and 22.99 g/100 g on DM, respectively (Table 3). The average ratio gelatinized/total starch was 0.68. Fiber fractions were 17.48, 4.67, and 1.85 g/100 g DM for NDF, ADF, and ADL, respectively (Table 3). Variation of starch and gelatinized starch among samples was around 25%, while coefficient of variation (CV) of the fiber fractions ranged from 37% (NDF) to 57% (ADF).

### 3.2. Near-Infrared Spectrum and Predictions Models

**Figure 1:**
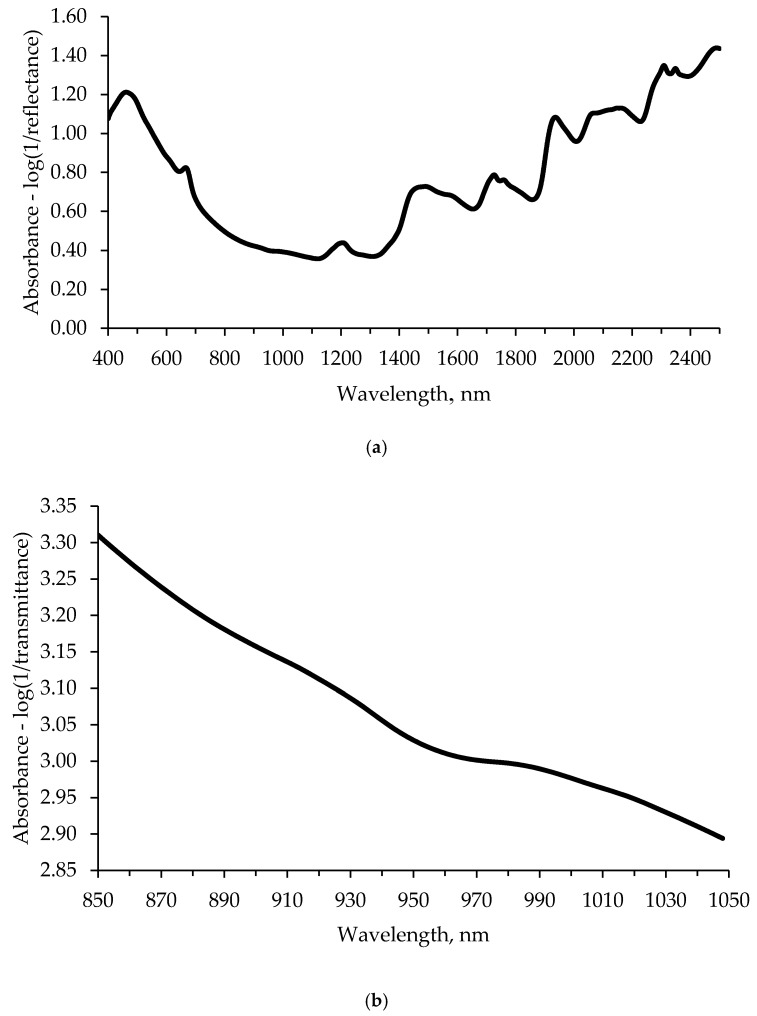
Average raw spectra of dry dog pet food using (**a**) visible/near-infrared spectroscopy in reflectance mode and (**b**) near-infrared spectroscopy in transmittance mode.

The average raw absorbance spectrum of the samples obtained with the two instruments are depicted in Figure 1. The Vis-NIR raw spectrum revealed sharp peaks at 465, 663, 1202, 1570, 1726, 1755, 1935, 2307, and 2348 nm and two wide peaks from 1454 to 1495 nm and from 2060 to 2168 nm (Figure 1a). On the other hand, NIT raw spectrum followed a quite fast-decreasing curve (Figure 1b).

The performance of the best prediction models for each instrument using the complete dataset are reported in Table 4, whereas using subsets for external validation are reported in Table 5. Outliers detected for the complete dataset were ≤9% for all the variables, except for the total starch predicted by Vis-NIR and ADF predicted by NIT (14% and 11% of samples deleted, respectively). When performing external validation (Table 5), outliers detected were ≤10% for all the variables, except for NDF predicted by NIT (15%). With the complete dataset (Table 4), LF ranged from 8 to 10 in both instruments. When performing external validation (Table 5), LF ranged from 7 to 10 for Vis-NIR and from 6 to 10 for NIT.

The best prediction models for Vis-NIR with the complete dataset (Table 4) were obtained when using SNV + D, MSC, WMSC, or ISC as the scatter correction and the first or second derivatives as the mathematical pretreatment. On the other hand, for NIT with the complete dataset (Table 4), the best prediction models were achieved without scatter correction (NONE) or D and with raw spectra or the first derivative. The best prediction models for Vis-NIR performing an external validation (Table 5) were obtained using MSC, WMSC, or ISC preprocessing techniques to reduce the variability due to scatter and using the first and second derivatives as the mathematical pretreatment. On the other hand, for NIT with external validation (Table 5), the best prediction models were obtained with NONE, D, or ISC, with raw spectra, or with the first or second derivatives.

For the complete dataset (Table 4), all prediction models for Vis-NIR achieved an RPD_CrV_ > 3.5, being the best the one for total starch (R^2^_CrV_ = 0.97; RPD_CrV_ = 6.33) and the worst for ADL (R^2^_CrV_ = 0.93; RPD_CrV_ = 3.73). On the other hand, considering the complete dataset (Table 4), RPD_CrV_ of the prediction models with NIT ranged from 1.75 (ADF) to 2.61 (ADL). When performing an external validation (Table 5), all prediction models for Vis-NIR achieved an RPD_ExV_ > 4, with the exception of ADL, being the best equation the one for ADF (R^2^_ExV_ = 0.97; RPD_ExV_ = 5.67) and the worst for ADL (R^2^_ExV_ = 0.84; RPD_ExV_ = 2.46). On the other hand, in external validation (Table 5), RPD_ExV_ of the prediction models obtained with NIT ranged from 1.71 (ADL) to 2.16 (total starch). Residuals of prediction equations obtained performing cross- and external validations were normally distributed, and bias did not differ statistically from zero.

## 4. Discussion

### 4.1. Samples Composition

Gross composition (Table 2) of the samples was consistent with the European Pet Food Industry Federation (FEDIAF) [29] recommendations. Moreover, total starch and fiber fractions variability, as well as the ingredients used, reflects the variability of food in the market connected to the formulations performed to meet the requirements of animals of different life stages and sizes.

Total starch content (Table 3) in the present study was consistent with those reported by several authors who determined this parameter in dry dog food using a reference analysis [5,30,31,32]. The ADF content (Table 3) agreed with results from De-Oliveira et al. [5] and Tran et al. [31]. The gelatinized starch content (Table 3) and the ratio gelatinized/total starch were slightly lower than the ones reported by Tran et al. [31]. In particular, the latter author reported a proportion of gelatinized starch of 0.76 in extruded dog food, which may be due to a slightly lower content of fat (9.7 g/100 g DM) in their samples, since fat content during extrusion has an inverse effect on the starch gelatinization degree [33]. However, in dog food, fats are usually added after the product is extruded, and differences on dog food composition (other than fat) and the processing condition may have contributed to the differences observed. Moreover, we obtained a greater NDF content than De-Oliveira et al. [5], who reported an average of 8.5% of NDF (DM basis) with a lower CV. The greater variability obtained in the present study is a consequence of the high variability of samples included, which is a key point to developing robust calibration models [23,24]. The ADL content (Table 3) was greater than the one reported by Opitz et al. [34] in extruded dog food but lower than that reported by Cipollini [35], who quantified ADL in the same matrix.

### 4.2. Vis-NIR vs. NIT Prediction Models Accuracy

The average raw absorbance spectrum for Vis-NIR (Figure 1a) is consistent with the one reported in other studies with ground dog food samples [23,36,37], even if they did not consider the visible region of the spectrum. The absorption signals observed at 1202 and 2348 nm are related to the presence of carbohydrates [38,39]; signals around 1460 and 1935 nm to water [39,40]; absorption signals at 1490 and 1570 nm to protein [40,41]; at 1726, 1755, and 2308 nm to lipids [17]; and at approximately 1450 and 2100 nm to starch [40]. On the other hand, NIT raw spectrum followed a decreasing curve, and no clear peaks were observed (Figure 1b). However, the NIT spectra agreed with the absorbance observed in the Vis-NIR spectra (Figure 1a) on the wavelength range from 850 to 1050 nm. The feasibility of near-infrared spectroscopy to evaluate pet food general composition has been assessed by several authors [18,19,23,36,37,42], but studies to predict total starch, damaged or gelatinized starch, NDF, ADF, and ADL in pet food are lacking.

Fitting statistics of the best prediction models were similar for cross- (Table 4) and external validations (Table 5) when using the same near-infrared spectroscopy instrument. Prediction models obtained by Vis-NIR technology with cross-validation can be considered good for NDF and ADL (RPD between 3.0 and 4.0) and excellent (RPD > 4.0) for total starch, gelatinized starch, and ADF. In compound feed for swine, a similar R^2^_CrV_ was obtained for total starch using a near-infrared instrument working between 1100 and 2500 nm [22]. However, we obtained better R^2^_CrV_ for fiber fractions than the cited authors (0.92, 0.95, and 0.85 for NDF, ADF, and ADL, respectively) [22]. When prediction models for Vis-NIR were tested with an external validation, predictions were considered as excellent for all the traits except for ADL, in which the RPD was below 2.5. In compound feed for rabbits, a lower R^2^_ExV_ has been reported for total starch, NDF, ADF, and ADL (0.90, 0.50, 0.82, and 0.59, respectively) using a near-infrared instrument that worked between 1100 and 2500 nm [21] in comparison to those obtained in the present study.

On the other hand, prediction models using NIT were, in general, unsatisfactory (RPD_CrV_ or RPD_ExV_ < 2.5; Table 4 and Table 5, respectively). Only when using cross-validation, prediction models achieved for total starch and ADL were adequate for screening. The slight difference between the fitting statistics obtained for cross- and external validations affected the interpretation of the model accuracy for some traits, because that interpretation is based on pre-established thresholds [43]. However, those changes were in agreement with the results obtained in the prediction of fatty acids and minerals in cheese when comparing cross- and external validations [44]. Although both procedures (cross- and external validations) are accepted for chemometric analysis, results obtained with external validation better reflects the prediction ability of the model for future samples [45].

Overall, Vis-NIR resulted to be better than NIT to predict total starch, gelatinized starch, and fiber fractions in extruded dry dog food. The better performance of infrared in reflectance than in transmittance mode has been already reported in meat [46] and fresh grass silage quality traits [47]; despite that, this was not observed when predicting minerals in fresh cheese [48]. Considering Figure 1, the greater accuracy of Vis-NIR technology is probably due to the extent of the range of the wavelength that provided a more informative spectrum in the first case rather than in the second one.

## 5. Conclusions

The results obtained demonstrated that total and gelatinized starch, as well as NDF, ADF, and ADL, can be successfully predicted with Vis-NIR spectroscopy. On the other hand, prediction models when using NIT spectroscopy presented low accuracy, which suggests that this instrument is not adequate to predict starch and fiber fractions in extruded dry dog food. A prediction model to determine gelatinized starch during the manufacture of dry dog food could be useful to monitor its proportion with respect to total starch. Moreover, to the best of our knowledge, this is the first time that near-infrared prediction models have been developed to determine gelatinized starch in pet food. Considering the good prediction models for total starch, gelatinized starch, NDF, ADF, and ADL, further studies are needed to confirm the feasibility of Vis-NIR to perform on-line or at-line quality control.

## Figures and Tables

**Table 1:** Brief description of the commercial dry dog food samples included in the study.

Main Carbohydrates Source	Main Protein Sources	Dog Size	Life Stage	Number of Packages
Oats	only chicken	medium	adult	2
Corn	chicken, duck, fish, lamb, rabbit	small, medium, medium/large, large	puppy, adult	25
Potato	chicken, duck, horse, rabbit venison	small, medium/large	puppy, adult	14
Pea	chicken, pork	small, medium/large	adult	14
Rice	fish, lamb, pork	small, medium, medium/large, large	adult	13
Sorghum	chicken, pork	small, medium, medium/large, large	puppy, adult	13

**Table 2:** Chemical composition (g/100 g dry matter (DM)) of the commercial dry dog food samples (*n* = 81) included in the study reported by the manufacturer.

Trait	Mean	SD	Minimum	Maximum	CV
Dry matter	92.00	0.00	92.00	92.00	0.00
Crude protein	28.98	4.68	23.91	40.22	16.10
Crude fats	15.90	3.45	10.33	21.74	21.71
Crude Fibers	3.62	2.59	2.28	15.22	71.56
Crude Ash	7.32	1.34	2.50	9.78	18.34

SD = standard deviation and CV = coefficient of variation; %.

**Table 3:** Chemical composition (g/100 g DM) obtained by reference analyses of dry dog food samples (*n* = 81) included in this study.

Trait	Mean	SD	Minimum	Maximum	CV
Total starch	34.22	7.64	11.87	46.77	22.3
Gelatinized starch	22.99	6.20	9.49	36.98	27.0
NDF	17.48	6.52	8.21	37.74	37.3
ADF	4.67	2.65	2.28	17.39	56.7
ADL	1.85	0.82	0.43	4.58	44.3

ADF = acid detergent fiber, ADL = acid detergent lignin, CV = coefficient of variation, NDF = neutral detergent fiber, and SD = standard deviation.

**Table 4:** Fitting statistics of modified partial least square regression models using one-leave-out cross-validation (*n* = 81) for total and gelatinized starch, neutral detergent fiber (NDF), acid detergent fiber (ADF), and acid detergent lignin (ADL) content (g/100 g DM) in extruded dry dog food with visible and near-infrared reflectance spectroscopy (Vis-NIR) and near-infrared transmittance spectroscopy (NIT).

Trait	LF	Mean	SD	R^2^_C_	SE_C_	R^2^_CrV_	SE_CrV_	RPD_CrV_
Vis-NIR, 400–2500 nm								
Total starch	8	34.89	7.34	0.99	0.59	0.97	1.16	6.33
Gelatinized starch	9	23.04	6.10	0.98	0.89	0.95	1.32	4.62
NDF	10	16.81	5.97	0.97	1.03	0.93	1.52	3.93
ADF	9	4.53	2.67	0.99	0.26	0.97	0.46	5.80
ADL	9	1.81	0.82	0.98	0.11	0.93	0.22	3.73
NIT, 850–1050 nm								
Total starch	8	34.84	7.02	0.88	2.42	0.84	2.77	2.53
Gelatinized starch	10	22.82	6.15	0.83	2.55	0.77	2.94	2.09
NDF	9	17.46	6.55	0.89	2.18	0.80	2.90	2.26
ADF	10	4.10	1.21	0.80	0.54	0.67	0.69	1.75
ADL	10	1.89	0.81	0.88	0.28	0.85	0.31	2.61

LF = optimal number of latent factors, R^2^_C_ = coefficient of determination of calibration, R^2^_CrV_ = coefficient of determination of cross-validation, RPD_CrV_ = residual predictive deviation of cross-validation, SD = standard deviation, SE_C_ = standard error of calibration, and SE_CrV_ = standard error of cross-validation.

**Table 5:** Fitting statistics of modified partial least square regression models using external validation for total and gelatinized starch, neutral detergent fiber (NDF), acid detergent fiber (ADF), and acid detergent lignin (ADL) content (g/100 g DM) in extruded dry dog food with visible/near-infrared reflectance spectroscopy (Vis-NIR) and near-infrared transmittance spectroscopy (NIT).

Trait	Calibration Set (*n* = 60)			Validation Set (*n* = 21)				
	LF	SE_CrV_	R^2^_CrV_	Bias	Slope	SE_P_	R^2^_ExV_	RPD_ExV_
Vis-NIR, 400–2500 nm								
Total starch	6	1.31	0.97	−0.29	0.90	1.60	0.96	4.43
Gelatinized starch	9	1.60	0.93	0.36	1.03	1.58	0.95	4.36
NDF	10	1.68	0.91	−1.12	1.04	1.63	0.95	4.31
ADF	10	0.54	0.95	−0.08	1.03	0.60	0.97	5.67
ADL	10	0.23	0.89	−0.04	1.02	0.28	0.84	2.46
NIT, 850–1050 nm								
Total starch	9	2.91	0.83	−0.16	0.89	3.28	0.80	2.16
Gelatinized starch	10	3.16	0.71	0.42	0.91	3.77	0.71	1.83
NDF	8	2.64	0.78	0.11	0.96	2.90	0.74	1.97
ADF	6	0.66	0.70	−0.18	0.77	0.72	0.75	1.77
ADL	9	0.26	0.83	−0.12	0.77	0.38	0.72	1.71

LF = latent factors, R^2^_ExV_ = coefficient of determination of external validation, RPD_ExV_ = residual predictive deviation of external validation, SD = standard deviation, SE_CrV_ = standard error of cross-validation, and SE_P_ = standard error of external validation.

## Supplementary Materials

The following are available online at https://www.mdpi.com/2076-2615/10/5/862/s1: Table S1. List of ingredients for each sample as declared by the manufacturer on the label of the product. Table S2. Chemical composition of each sample as declared by the manufacturer on the label of the product.
