# Supplementary material for: At-line Prediction of Gelatinized Starch and Fiber Fractions in Extruded Dry Dog Food Using Different Near-Infrared Spectroscopy Technologies
Source: Animals (Basel). 2020 May 16;10(5):862. doi: 10.3390/ani10050862 (PMC7278468; doi:10.3390/ani10050862)
Supplement: Supplementary file 1 [file animals-10-00862-s001.pdf]

Table S1 - List of ingredients for each sample as declared by the manufacturer in the label of the product

sample	ingredients
1	dehydrated chicken (26%), sorghum (20%), pea, chicken fat, dehydrated pork (9%), dehydrated alfalfa, dehydrated salmon (6%), hydrolyzed chicken protein, dehydrated whole eggs, salmon oil (1%), yeast, dried chicory pulp, sodium chloride, dehydrated turmeric (0.3%), fructo-oligosaccharides (0.2%), mannan-oligosaccharides (0.2%), glucosamine (0.15%), chondroitin sulphate (0.09%), red raspberry dehydrated (0.05%), dehydrated blackberry, dehydrated blackcurrant, pomegranate dehydrated, mojave yucca
2	dehydrated chicken (25%), sorghum (20%), pea, chicken fat, dehydrated pork (9%), dehydrated alfalfa, dehydrated salmon (6%), hydrolyzed chicken protein, dehydrated whole eggs, yeast, dried chicory pulp, salmon oil (1%), sodium chloride, dehydrated turmeric (0.3%), fructo-oligosaccharides (0.2%), mannan-oligosaccharides (0.2%), glucosamine (0.15%), calcium carbonate, chondroitin sulphate (0.09%), red raspberry dehydrated (0.05%), dehydrated blackberry, dehydrated blackcurrant, pomegranate dehydrated, mojave yucca
3	dehydrated chicken (26%), sorghum (20%), pea, chicken fat, dehydrated pork (9%), dehydrated alfalfa, dehydrated salmon (6%), hydrolyzed chicken protein, dehydrated whole eggs, salmon oil (1%), yeast, dried chicory pulp, sodium chloride, dehydrated turmeric (0.3%), fructo-oligosaccharides (0.2%), mannan-oligosaccharides (0.2%), glucosamine (0.15%), chondroitin sulphate (0.09%), red raspberry dehydrated (0.05%), dehydrated blackberry, dehydrated blackcurrant, pomegranate dehydrated, mojave yucca
4	dehydrated chicken (26%), sorghum (20%), pea, chicken fat, dehydrated pork (9%), dehydrated alfalfa, dehydrated salmon (6%), hydrolyzed chicken protein, dehydrated whole eggs, salmon oil (1%), yeast, dried chicory pulp, sodium chloride, dehydrated turmeric (0.3%), fructo-oligosaccharides (0.2%), mannan-oligosaccharides (0.2%), glucosamine (0.15%), chondroitin sulphate (0.09%), red raspberry dehydrated (0.05%), dehydrated blackberry, dehydrated blackcurrant, pomegranate dehydrated, mojave yucca
5	dehydrated chicken (25%), sorghum (20%), pea, chicken fat, dehydrated pork (9%), dehydrated alfalfa, dehydrated salmon (6%), hydrolyzed chicken protein, dehydrated whole eggs, yeast, dried chicory pulp, salmon oil (1%), sodium chloride, dehydrated turmeric (0.3%), fructo-oligosaccharides (0.2%), mannan-oligosaccharides (0.2%), glucosamine (0.15%), calcium carbonate, chondroitin sulphate (0.09%), red raspberry dehydrated (0.05%), dehydrated blackberry, dehydrated blackcurrant, pomegranate dehydrated, mojave yucca
6	dehydrated pork (37%), dehydrated pea, dehydrated banana, chicken fat, dehydrated rabbit (5%), hydrolyzed chicken protein, dehydrated alfalfa, salmon oil, dehydrated herring, dehydrated whole eggs (1%), yeast, dried chicory pulp, sodium chloride, calcium carbonate, linseed oil (0.4%), fructo-oligosaccharides (0.2%), mannan-oligosaccharides (0.2%), apple, pomegranate, pumpkin, green tea, spinach, fennel, borage, chamomile, liquorice, tomato, rosehip, sage, peppermint, glucosamine (0.04%), raspberry, blackberry, mojave yucca, chondroitin sulphate (0.01%)
7	dehydrated chicken (42%), dehydrated pea, dehydrated banana, chicken fat, dehydrated duck (6%), hydrolyzed chicken protein, dehydrated alfalfa, salmon oil, dehydrated herring, dehydrated whole eggs (1%), yeast, dried chicory pulp, sodium chloride, monosodium phosphate, linseed oil (0.4%), fructo-oligosaccharides (0.2%), mannan-oligosaccharides (0.2%), apple, pomegranate, pumpkin, green tea, spinach, fennel, borage, chamomile, liquorice, tomato, rosehip, sage, peppermint, glucosamine (0.04%), raspberry, blackberry, mojave yucca, chondroitin sulphate (0.01%)
8	dehydrated chicken (26%), sorghum (20%), pea, dehydrated alfalfa, dehydrated pork (7%), dehydrated salmon (7%), dried chicory pulp, hydrolyzed chicken protein, chicken fat, salmon oil (1%), yeast, dehydrated whole eggs, sodium chloride, dehydrated turmeric (0.3%), fructo-oligosaccharides (0.2%), mannan-oligosaccharides (0.2%), glucosamine (0.15%), chondroitin sulphate (0.09%), red raspberry dehydrated (0.05%), dehydrated blackberry, dehydrated blackcurrant, pomegranate dehydrated, mojave yucca
9	rice (49%), dehydrated pork (26%), pork fat, dried chicory pulp, dehydrated alfalfa, hydrolyzed pork protein, monocalcic phosphate, brewer's yeast (3%), potassium chloride, pea fiber, flax seeds, sodium chloride, mannan-oligosaccharides (0,3%), fructo-oligosaccharides (0.2%), psyllium husks and seeds, β-1,3/1,6 glucans from baker's yeast Saccaromyces cerevisiae (0.07%)
10	dehydrated chicken (22%), sorghum (20%), pea, chicken fat, dehydrated pork (8%), dehydrated alfalfa, dehydrated salmon (6%), hydrolyzed chicken protein, yeast, dried chicory pulp, salmon oil (0.8%), dehydrated whole eggs, sodium chloride, dehydrated turmeric (0.3%), fructo-oligosaccharides (0.2%), mannan-oligosaccharides (0.2%), calcium carbonate, glucosamine (0.15%), chondroitin sulphate (0.09%), red raspberry dehydrated (0.05%), dehydrated blackberry, dehydrated blackcurrant, pomegranate dehydrated, mojave yucca

11	pea (65%), dehydrated pork (22%), sunflower oil, hydrolyzed pork protein, flax seeds (1%), calcium carbonate, sodium chloride, aloe vera (0.03%), rosemary
12	rice (51%), dehydrated pork (25%), pork fat, dried chicory pulp, dehydrated alfalfa, monocalcic phosphate, brewer's yeast (3%), potassium chloride, pea fiber, flax seeds, sodium chloride, mannan-oligosaccharides (0,3%), fructo-oligosaccharides (0.2%), psyllium husks and seeds, β-1,3/1,6 glucans from baker's yeast Saccaromyces cerevisiae (0.07%)
13	potato (70%), dehydrated rabbit (20%), sunflower oil, calcium carbonate, monosodium phosphate, flax seeds (1%), sodium chloride, aloe vera (0.03%), rosemary
14	dehydrated pork (22%), sorghum (20%), pea, dehydrated lamb (10%), pork fat, dehydrated alfalfa, hydrolyzed pork protein, dehydrated whole eggs (1%), yeast, dried chicory pulp, salmon oil (0.8%), sodium chloride, dehydrated turmeric (0.3%), fructo-oligosaccharides (0.2%), mannan-oligosaccharides (0.2%), glucosamine (0.15%), chondroitin sulphate (0.09%), red raspberry dehydrated (0.05%), dehydrated blackberry, dehydrated blackcurrant, pomegranate dehydrated, mojave yucca
15	dehydrated lamb (28%), rice, pea, pork fat, hydrolysed animal proteins with low molecular weight, dehydrated alfalfa, hemp (1%), yeast, dried chicory pulp (0.5%), sodium chloride, camelina oil (0.3%), olive oil (0.3%), β-1,3 glucans from baker's yeast Saccaromyces cerevisiae (0.06%), glucosamine (0.04%), dehydrated broccoli (0.03%), pomegranate dehydrated (0.03%), tomato dehydrated (0.03%), mojave yucca, chondroitin sulphate (0.01%)
16	dehydrated chicken (30%), corn, rice, chicken fat, dried beet pulp, hydrolyzed chicken protein, flax seeds oil (1.4%), yeast, olive oil (0.4%), dried chicory pulp (0,4%), pea fiber (0.4%), calcium carbonate, sodium chloride, glucosamine (0.04%), pomegranate dehydrated (0.03%), tomato dehydrated (0.03%), mojave yucca (0.02%), chondroitin sulphate (0.01%)
17	cereals (rice 4%), meat and animal derivatives (chicken 13%), oils and fats, derivatives of vegetable origin, minerals
18	potato (71%), dehydrated venison (20%), sunflower oil, flax seeds (1%), monosodium phosphate, sodium chloride, aloe vera (0.03%), rosemary
19	dehydrated duck (30%), corn, rice, chicken fat, dried beet pulp, yeast, hydrolyzed chicken protein, flax seeds oil (1.4%), olive oil (0,4%), dried chicory pulp (0.4%), pea fiber (0.4%), calcium carbonate, sodium chloride, pomegranate dehydrated (0.03%), tomato dehydrated (0.03%), mojave yucca (0.02%)
20	dehydrated chicken (30%), oats, corn, rice, dried beet pulp, dried chicory pulp (5%), chicken fat, yeast, hydrolyzed chicken protein, flax seeds oil (1.4%), pea fiber (1%), olive oil (0.4%), calcium carbonate, fructo-oligosaccharides (0.2%), sodium chloride, glucosamine, pomegranate dehydrated (0.03%), tomato dehydrated (0.03%), mojave yucca (0.02%)
21	Corn, dehydrated fish (22%), rice, chicken fat, dried beet pulp, flax seeds oil (1.4%), yeast, olive oil (0.4%), dried chicory pulp (0.4%), pea fiber (0.2%), calcium carbonate, sodium chloride, pomegranate dehydrated (0.03%), tomato dehydrated (0.03%), mojave yucca (0.02%)
22	Corn, dehydrated chicken (30%), rice, pork fat, dried beet pulp, hydrolyzed chicken protein, flax seeds oil (0.5%), dried chicory pulp (0.3%), pea fiber (0.4%), calcium carbonate, sodium chloride, glucosamine (0.04%), pomegranate dehydrated (0.03%), tomato dehydrated (0.03%), mojave yucca (0.02%), chondroitin sulphate (0.01%)
23	dehydrated lamb (28%), rice, pea, pork fat, hydrolysed animal proteins with low molecular weight, dehydrated alfalfa, hemp (1%), yeast, dried chicory pulp (0.5%), sodium chloride, camelina oil (0.3%), olive oil (0.3%), β-1,3 glucans from baker's yeast Saccaromyces cerevisiae (0.06%), glucosamine (0.04%), dehydrated broccoli (0.03%), pomegranate dehydrated (0.03%), tomato dehydrated (0.03%), mojave yucca, chondroitin sulphate (0.01%)
24	dehydrated chicken (32%), corn, rice, chicken fat, dried beet pulp, hydrolyzed chicken protein, flax seeds oil (1.4%), yeast, olive oil (0.4%), dried chicory pulp (0.4%), pea fiber (0.4%), calcium carbonate, sodium chloride, glucosamine (0.04%), pomegranate dehydrated (0.03%), tomato dehydrated (0.03%), mojave yucca (0.02%)
25	dehydrated lamb (28%), rice, pea, pork fat, hydrolysed animal proteins with low molecular weight, dehydrated alfalfa, hemp (1%), yeast, dried chicory pulp (0,5%), sodium chloride, camelina oil (0.3%), olive oil (0.3%), β-1,3 glucans from baker's yeast Saccaromyces cerevisiae (0.06%), glucosamine (0.04%), dehydrated broccoli (0.03%), pomegranate dehydrated (0.03%), tomato dehydrated (0.03%), mojave yucca, chondroitin sulphate (0.01%)
26	dehydrated chicken (32%), corn, rice, chicken fat, dried beet pulp, hydrolyzed chicken protein, flax seeds oil (1.4%), yeast, olive oil (0.4%), dried chicory pulp (0.4%), pea fiber (0.4%), calcium carbonate, sodium chloride, glucosamine (0.04%), pomegranate dehydrated (0.03%), tomato dehydrated (0.03%), mojave

	yucca (0.02%)
27	Corn, dehydrated fish (20%), rice, chicken fat, dried beet pulp, flax seeds oil (1.4%), yeast, olive oil (0.4%), pea fiber (0.4%), dried chicory pulp (0.4%), calcium carbonate, sodium chloride, pomegranate dehydrated (0.03%), tomato dehydrated (0.03%), mojave yucca (0.02%)
28	dehydrated lamb (25%), rice, corn, pork fat, dehydrated alfalfa, dried beet pulp, yeast, sodium chloride, monosodium phosphate, camelina oil (0.3%), olive oil (0.3%), β-1,3 glucans from baker's yeast Saccaromyces cerevisiae (0.06%), glucosamine (0.04%), dehydrated broccoli (0.03%), pomegranate dehydrated (0,03%), tomato dehydrated (0.03%), mojave yucca, chondroitin sulphate (0.01%)
29	dehydrated duck (30%), corn, rice, chicken fat, dehydrated alfalfa, flax seeds oil (1.4%), yeast, olive oil (0.4%), dried chicory pulp (0.4%), pea fiber (0.4%), calcium carbonate, sodium chloride, pomegranate dehydrated (0.03%), tomato dehydrated (0.03%), mojave yucca (0.02%)
30	Corn, dehydrated fish (20%), rice, chicken fat, dried beet pulp, flax seeds oil (1.4%), yeast, olive oil (0.4%), dried chicory pulp (0.4%), pea fiber (0.4%), sodium chloride, pomegranate dehydrated (0.03%), tomato dehydrated (0,03%), mojave yucca (0.02%)
31	dehydrated chicken (30%), corn, rice, chicken fat, dried beet pulp, hydrolyzed chicken protein, sodium chloride, flax seeds oil (1.4%), yeast, olive oil (0.4%), dried chicory pulp (0.4%), pea fiber (0.4%), calcium carbonate, glucosamine (0.04%), pomegranate dehydrated (0.03%), tomato dehydrated (0.03%), mojave yucca (0.02%)
32	dehydrated rabbit (23%), corn, rice, chicken fat, dried beet pulp, yeast, flax seeds oil (1.4%), olive oil (0.4%), dried chicory pulp (0.4%), pea fiber (0.4%), calcium carbonate, glucosamine (0.04%), pomegranate dehydrated (0.03%), tomato dehydrated (0.03%), mojave yucca (0.02%)
33	dehydrated lamb (25%), rice, corn, pork fat, dehydrated alfalfa, dried beet pulp, yeast, sodium chloride, monosodium phosphate, camelina oil (0.3%), olive oil (0.3%), β-1,3 glucans from baker's yeast Saccaromyces cerevisiae (0.06%), glucosamine (0.04%), dehydrated broccoli (0.03%), pomegranate dehydrated (0.03%), tomato dehydrated (0.03%), mojave yucca, chondroitin sulphate (0.01%)
34	dehydrated chicken (30%), corn, rice, chicken fat, dried beet pulp, hydrolyzed chicken protein, sodium chloride, flax seeds oil (1.4%), yeast, olive oil (0.4%), dried chicory pulp (0.4%), pea fiber (0.4%), calcium carbonate, glucosamine (0.04%), pomegranate dehydrated (0.03%), tomato dehydrated (0.03%), mojave yucca (0.02%)
35	potato (68%), dehydrated duck (21%), sunflower oil, flax seeds (1%), monosodium phosphate, sodium chloride, aloe vera (0.03%), rosemary
36	Dehydrated fish (28%), corn, rice, chicken fat, dried beet pulp, dehydrated alfalfa, yeast, monosodium phosphate, flax seeds oil (0.5%), dried chicory pulp (0.3%), olive oil (0.3%), fructo-oligosaccharides, mannan-oligosaccharides (0.04%), glucosamine (0.04%), red pomegranate dehydrated (0.03%), tomato dehydrated (0.03%), mojave yucca, chondroitin sulphate (0.01%)
37	dehydrated chicken (32%), corn, rice, chicken fat, dried beet pulp, dehydrated alfalfa, hydrolysed chicken protein, yeast, sodium chloride, monosodium phosphate, flax seeds oil (0.5%), dried chicory pulp (0.3%), olive oil (0.3%), fructo-oligosaccharides, β-1,3/1,6 glucans from baker's yeast Saccaromyces cerevisiae (0.04%), , glucosamine (0.04%), pomegranate dehydrated (0.03%), tomato dehydrated (0.03%), mojave yucca (0.02%), chondroitin sulphate (0.01%)
38	dehydrated pork (20%), sorghum, pea, wood substances, dried beet pulp, hemp (4%), dried chicory pulp, dehydrated alfalfa, pork fat, oat fibre, salmon oil, hydrolyzed pork protein, yeast, sodium chloride, calcium carbonate, β-1,3/1,6 glucans from baker's yeast Saccaromyces cerevisiae (0.1%), glucosamine (0.06%), chondroitin sulphate (0.03%)
39	dehydrated chicken (22%), sorghum (20%), pea, chicken fat, dehydrated pork (7%), dehydrated salmon (6%), dehydrated alfalfa, hydrolyzed chicken protein, yeast, dried chicory pulp, salmon oil (0.8%), dehydrated whole eggs, sodium chloride, dehydrated turmeric (0.3%), fructo-oligosaccharides (0.2%), mannan-oligosaccharides (0.2%), calcium carbonate, glucosamine (0.15%), chondroitin sulphate (0.09%), red raspberry dehydrated (0.05%), dehydrated blackberry, dehydrated blackcurrant, pomegranate dehydrated, mojave yucca
40	Corn, dehydrated fish (20%), rice, chicken fat, dried beet pulp, flax seeds oil (1.4%), yeast, olive oil (0.4%), dried chicory pulp (0.4%), pea fiber (0.4%), sodium chloride, pomegranate dehydrated (0.03%), tomato dehydrated (0.03%), mojave yucca (0.02%)
41	dehydrated chicken (31%), corn, rice, chicken fat, dried beet pulp, yeast, dehydrated whole eggs, hydrolyzed chicken protein, flax seeds oil (1.4%), olive oil (0.4%), dried chicory pulp (0.4%), pea fiber (0.4%), calcium carbonate, sodium chloride, fructo-oligosaccharides (0.2%), glucosamine (0.04%), pomegranate dehydrated (0.03%), tomato dehydrated (0.03%), mojave yucca (0.02%), chondroitin sulphate (0.01%)

42	dehydrated chicken (32%), corn, rice, chicken fat, dried beet pulp, yeast, hydrolyzed chicken protein, dehydrated whole eggs, flax seeds oil (1.4%), olive oil (0.4%), pea fiber (0.4%), dried chicory pulp (0.4%), calcium carbonate, sodium chloride, fructo-oligosaccharides (0.2%), pomegranate dehydrated (0.03%), tomato dehydrated (0.03%), mojave yucca (0.02%)
43	rice (51%), dehydrated pork (25%), pork fat, dried chicory pulp, dehydrated alfalfa, monocalcic phosphate, brewer's yeast (3%), potassium chloride, pea fiber, flax seeds, sodium chloride, mannan-oligosaccharides (0.3%), fructo-oligosaccharides (0.2%), psyllium husks and seeds, β-1,3/1,6 glucans from baker's yeast Saccaromyces cerevisiae (0.07%)
44	dehydrated chicken (31%), corn, rice, chicken fat, dried beet pulp, yeast, dehydrated whole eggs, hydrolyzed chicken protein, flax seeds oil (1.4%), olive oil (0.4%), dried chicory pulp (0.4%), pea fiber (0.4%), calcium carbonate, sodium chloride, fructo-oligosaccharides (0.2%), glucosamine (0.04%), pomegranate dehydrated (0.03%), tomato dehydrated (0.03%), mojave yucca (0.02%), chondroitin sulphate (0.01%)
45	Dehydrated fish (29%), corn, rice, chicken fat, dried beet pulp, dehydrated alfalfa, yeast, monosodium phosphate, flax seeds oil (0.5%), dried chicory pulp (0.3%), olive oil (0.3%), fructo-oligosaccharides, mannan-oligosaccharides (0.04%), glucosamine (0.04%), red pomegranate dehydrated (0.03%), tomato dehydrated (0.03%), mojave yucca
46	potato (68%), dehydrated duck (21%), sunflower oil, flax seeds (1%), monosodium phosphate, sodium chloride, aloe vera (0.03%), rosemary
47	dehydrated chicken (32%), corn, rice, chicken fat, dried beet pulp, dehydrated alfalfa, hydrolysed chicken protein, yeast, sodium chloride, monosodium phosphate, flax seeds oil (0.5%), dried chicory pulp (0.3%), olive oil (0.3%), fructo-oligosaccharides, β-1,3/1,6 glucans from baker's yeast Saccaromyces cerevisiae (0.04%), , glucosamine (0,04%), pomegranate dehydrated (0.03%), tomato dehydrated (0.03%), mojave yucca (0.02%), chondroitin sulphate (0.01%)
48	dehydrated chicken (32%), corn, rice, chicken fat, dried beet pulp, yeast, hydrolyzed chicken protein, dehydrated whole eggs, flax seeds oil (1.4%), olive oil (0.4%), pea fiber (0.4%), dried chicory pulp (0.4%), calcium carbonate, sodium chloride, fructo-oligosaccharides (0.2%), pomegranate dehydrated (0.03%), tomato dehydrated (0.03%), mojave yucca (0.02%)
49	potato (70%), dehydrated rabbit (20%), sunflower oil, calcium carbonate, monosodium phosphate, flax seeds (1%), sodium chloride, aloe vera (0.03%), rosemary
50	potato (70%), dehydrated horse (19%), sunflower oil, flax seeds (1%), sodium chloride, aloe vera (0.03%), rosemary
51	potato (70%), dehydrated rabbit (20%), sunflower oil, calcium carbonate, monosodium phosphate, flax seeds (1%), sodium chloride, aloe vera (0.03%), rosemary
52	dehydrated chicken (26%), sorghum (20%), pea, chicken fat, dehydrated pork (9%), dehydrated alfalfa, dehydrated salmon (6%), hydrolyzed chicken protein, dehydrated whole eggs, salmon oil (1%), yeast, dried chicory pulp, sodium chloride, dehydrated turmeric (0.3%), fructo-oligosaccharides (0.2%), mannan-oligosaccharides (0.2%), glucosamine (0.15%), chondroitin sulphate (0.09%), red raspberry dehydrated (0.05%), dehydrated blackberry, dehydrated blackcurrant, pomegranate dehydrated, mojave yucca
53	Dehydrated fish (28%), corn, rice, chicken fat, dried beet pulp, dehydrated alfalfa, yeast, monosodium phosphate, flax seeds oil (0.5%), dried chicory pulp (0.3%), olive oil (0.3%), fructo-oligosaccharides, mannan-oligosaccharides (0.04%), glucosamine (0.04%), red pomegranate dehydrated (0.03%), tomato dehydrated (0.03%), mojave yucca, chondroitin sulphate (0.01%)
54	dehydrated pork (37%), dehydrated pea, dehydrated banana, chicken fat, dehydrated rabbit (5%), hydrolyzed chicken protein, dehydrated alfalfa, salmon oil, dehydrated herring, dehydrated whole eggs (1%), yeast, dried chicory pulp, sodium chloride, calcium carbonate, linseed oil (0.4%), fructo-oligosaccharides (0.2%), mannan-oligosaccharides (0.2%), apple, pomegranate, pumpkin, green tea, spinach, fennel, borage, chamomile, liquorice, tomato, rosehip, sage, peppermint, glucosamine (0.04%), rasberry, blackberry, mojave yucca, chondroitin sulphate (0.01%)
55	dehydrated chicken (30%), oats, corn, rice, dried beet pulp, dried chicory pulp (5%), chicken fat, yeast, hydrolyzed chicken protein, flax seeds oil (1.4%), pea fiber (1%), olive oil (0.4%), calcium carbonate, fructo-oligosaccharides (0.2%), sodium chloride, glucosamine, pomegranate dehydrated (0.03%), tomato dehydrated (0.03%), mojave yucca (0.02%)
56	cereals (rice 4%), meat and animal derivatives (lamb 5%), oils and fats, derivatives of vegetable origin, minerals
57	cereals (rice 4%), meat and animal derivatives, oils and fats, fish and fish derivatives (5%), derivatives of vegetable origin, minerals
58	pea (65%), dehydrated pork (22%), sunflower oil, hydrolyzed pork protein, flax seeds (1%), calcium

	carbonate, sodium chloride, aloe vera (0.03%), rosemary
59	pea (65%), dehydrated pork (22%), sunflower oil, hydrolyzed pork protein, flax seeds (1%), calcium carbonate, sodium chloride, aloe vera (0.03%), rosemary
60	dehydrated pork (21%), sorghum, pea, wood substances, dried beet pulp, hemp (4%), dried chicory pulp, dehydrated alfalfa, pork fat, oat fibre, salmon oil, hydrolyzed pork protein, yeast, sodium chloride, calcium carbonate, β-1,3/1,6 glucans from baker's yeast Saccaromyces cerevisiae (0.1%), glucosamine (0.06%), chondroitin sulphate (0.03%)
61	dehydrated duck (33%), corn, rice, chicken fat, dried beet pulp, yeast, flax seeds oil (1.4%), olive oil (0.4%), dried chicory pulp (0.4%), pea fiber (0.4%), calcium carbonate, sodium chloride, pomegranate dehydrated (0.03%), tomato dehydrated (0.03%), mojave yucca (0.02%)
62	dehydrated lamb (22%), rice, corn, pork fat, dried beet pulp, yeast, flax seeds oil (1.4%), olive oil (0.4%), dried chicory pulp (0.4%), pea fiber (0.4%), calcium carbonate, sodium chloride, pomegranate dehydrated (0.03%), tomato dehydrated (0.03%), mojave yucca (0.02%)
63	potato (70%), dehydrated horse (19%), sunflower oil, flax seeds (1%), sodium chloride, aloe vera (0.03%), rosemary
64	potato (68%), dehydrated duck (21%), sunflower oil, flax seeds (1%), monosodium phosphate, sodium chloride, aloe vera (0.03%), rosemary
65	dehydrated pork (20%), sorghum, pea, wood substances, dried beet pulp, hemp (4%), dried chicory pulp, dehydrated alfalfa, pork fat, oat fibre, salmoin oil, hydrolyzed pork protein, yest, sodium chloride, calcium carbonate, β-1,3/1,6 glucans from baker's yeast Saccaromyces cerevisiae (0.1%), glucosamine (0.06%), chondroitin sulphate (0.03%)
66	dehydrated pork (37%), dehydrated pea, dehydrated banana, chicken fat, dehydrated rabbit (5%), hydrolyzed chicken protein, dehydrated alfalfa, salmon oil, dehydrated herring, dehydrated whole eggs (1%), yeast, dried chicory pulp, sodium chloride, calcium carbonate, linseed oil (0.4%), fructo-oligosaccharides (0.2%), mannan-oligosaccharides (0.2%), apple, pomegranate, pumpkin, green tea, spinach, fennel, borage, chamomile, liquorice, tomato, rosehip, sage, peppermint, glucosamine (0.04%), rasberry, blackberry, mojave yucca, chondroitin sulphate (0.01%)
67	dehydrated rabbit (23%), corn, rice, chicken fat, dried beet pulp, yeast, flax seeds oil (1.4%), olive oil (0,4%), dried chicory pulp (0.4%), pea fiber (0.4%), calcium carbonate, glucosamine (0.04%), pomegranate dehydrated (0.03%), tomato dehydrated (0.03%), mojave yucca (0.02%)
68	dehydrated rabbit (24%), corn, rice, chicken fat, dried beet pulp, dehydrated alfalfa, monosodium phosphate, sodium chloride, calcium carbonate, yeast, pomegranate dehydrated (0.03%), tomato dehydrated (0.03%), mojave yucca
69	dehydrated pork (37%), dehydrated pea, dehydrated banana, chicken fat, dehydrated rabbit (5%), hydrolyzed chicken protein, dehydrated alfalfa, salmoin oil, dehydrated herring, dehydrated whole eggs (1%), yest, dried chicory pulp, sodium chloride, calcium carbonate, linseed oil (0.4%), fructo-oligosaccharides (0.2%), mannan-oligosaccharides (0.2%), apple, pomegranate, pumpkin, green tea, spinach, fennel, borage, chamomile, liquorice, tomato, rosehip, sage, peppermint, glucosamine (0.04%), rasberry, blackberry, mojave yucca, chondroitin sulphate (0.01%)
70	potato (70%), dehydrated horse (19%), sunflower oil, flax seeds (1%), sodium chloride, aloe vera (0.03%), rosemary
71	pea (65%), dehydrated pork (22%), sunflower oil, hydrolyzed pork protein, flax seeds (1%), calcium carbonate, sodium chloride, aloe vera (0.03%), rosemary
72	pea (65%), dehydrated pork (22%), sunflower oil, hydrolyzed pork protein, flax seeds (1%), calcium carbonate, sodium chloride, aloe vera (0.03%), rosemary
73	pea (65%), dehydrated pork (22%), sunflower oil, hydrolyzed pork protein, flax seeds (1%), calcium carbonate, sodium chloride, aloe vera (0.03%), rosemary
74	potato (70%), dehydrated rabbit (20%), sunflower oil, calcium carbonate, monosodium phosphate, flax seeds (1%), sodium chloride, aloe vera (0.03%), rosemary
75	potato (70%), dehydrated rabbit (20%), sunflower oil, monosodium phosphat, calcium carbonate, flax seeds (1%), sodium chloride, aloe vera (0.03%), rosemary
76	dehydrated pork (37%), dehydrated pea, dehydrated banana, chicken fat, dehydrated rabbit (5%), hydrolyzed chicken protein, dehydrated alfalfa, salmoin oil, dehydrated herring, dehydrated whole eggs (1%), yest, dried chicory pulp, sodium chloride, calcium carbonate, linseed oil (0.4%), fructo-oligosaccharides (0.2%), mannan-oligosaccharides (0.2%), apple, pomegranate, pumpkin, green tea, spinach, fennel, borage, chamomile, liquorice, tomato, rosehip, sage, peppermint, glucosamine (0.04%), rasberry, blackberry, mojave yucca, chondroitin sulphate (0.01%)

77	potato (68%), dehydrated duck (21%), sunflower oil, flax seeds (1%), monosodium phosphate, sodium chloride, aloe vera (0.03%), rosemary
78	dehydrated lamb (28%), rice, pea, pork fat, hydrolysed animal proteins with low molecular weight, dehydrated alfalfa, hemp (1%), yeast, dried chicory pulp (0.5%), sodium chloride, camelina oil (0.3%), olive oil (0.3%), β-1,3 glucans from baker's yeast Saccaromyces cerevisiae (0.06%), glucosamine (0.04%), dehydrated broccoli (0,03%), pomegranate dehydrated (0.03%), tomato dehydrated (0.03%), mojave yucca, chondroitin sulphate (0.01%)
79	dehydrated pork (37%), dehydrated pea, dehydrated banana, chicken fat, dehydrated rabbit (5%), hydrolyzed chicken protein, dehydrated alfalfa, salmon oil, dehydrated herring, dehydrated whole eggs (1%), yeast, dried chicory pulp, sodium chloride, calcium carbonate, linseed oil (0.4%), fructo-oligosaccharides (0.2%), mannan-oligosaccharides (0.2%), apple, pomegranate, pumpkin, green tea, spinach, fennel, borage, chamomile, liquorice, tomato, rosehip, sage, peppermint, glucosamine (0.04%), rasberry, blackberry, mojave yucca, chondroitin sulphate (0.01%)
80	rice (49%), dehydrated pork (26%), pork fat, dried chicory pulp, dehydrated alfalfa, hydrolyzed pork protein, monocalcic phosphate, brewer's yeast (3%), potassium chloride, pea fiber, flax seeds, sodium chloride, mannan-oligosaccharides (0.3%), fructo-oligosaccharides (0.2%), psyllium husks and seeds, β-1,3/1,6 glucans from baker's yeast Saccaromyces cerevisiae (0.07%)
81	potato (70%), dehydrated horse (19%), sunflower oil, flax seeds (1%), sodium chloride, aloe vera (0.03%), rosemary

Table S2 - Chemical composition of each sample as declared by the manufacturer in the label of the product (g/100g as is)

sample	Crude Protein	Crude Fat	Crude Ash	Crude Fiber	Moisture
1	34	20	6.3	3.3	8
2	32	20	6	3.3	8
3	34	20	6.3	3.3	8
4	34	20	6.3	3.3	8
5	32	20	6	3.3	8
6	36	17	6	3.6	8
7	37	17	8	3.5	8
8	34	9.5	6.8	7	8
9	24	13	6.6	2.1	8
10	32	18.5	6	3.2	8
11	23.5	12	5	2.3	8
12	23	12	6.5	2.1	8
13	22	10	6.5	3.5	8
14	30	18	6	3.5	8
15	25	16	8	2.5	8
16	25	16	7	2.3	8
17	23	14	7.3	2.3	8
18	22	10	7	2.5	8
19	25.5	16	7.5	2.3	8
20	27	10.5	9	6.5	8
21	27	16	6.5	2.2	8
22	25	16	7	2.3	8
23	25	16	7	2.5	8
24	27	16	8	2.2	8
25	25	16	7	2.5	8
26	27	16	8	2.2	8
27	25	16	6.2	2.3	8
28	25	15	9	2.5	8
29	25	16	8	2.2	8
30	26	16	7.2	2.3	8
31	25	16	7	2.3	8
32	23	14.5	6.5	2.5	8
33	25	15	9	2.5	8
34	25	16	7	2.3	8
35	22.5	10	6.5	2.5	8
36	25	16	2.3	7	8
37	28	18	7	2.3	8
38	26	11	5.8	14	8
39	30	18	5.6	3.2	8
40	26	16	7.2	2.3	8
41	27	18	7	2.2	8
42	32	20	8.5	2.2	8
43	23	12	6.5	2.1	8
44	27	18	7	2.2	8
45	28	18	8	2.3	8
46	23.5	10	6.5	2.5	8
47	28	18	7	2.3	8
48	32	20	8.5	2.2	8
49	22	10	6.5	3.5	8
50	23.5	11	8.5	2.3	8
51	22	10	6.5	3.5	8

52	29	16.5	6	3.3	8
53	25	16	2.3	7	8
54	36	17	6	3.6	8
55	27	10.5	9	6.5	8
56	22.5	14	7.6	2.4	8
57	22.5	14	7.2	2.3	8
58	24.5	12	5	2.3	8
59	24.5	12	5	2.3	8
60	26.7	11	5.8	14	8
61	26.5	16	8	2.2	8
62	23	14	8	2.3	8
63	22.5	11	7.8	2.3	8
64	22.5	10	6.5	2.5	8
65	26	11	5.8	14	8
66	36	17	6	3.6	8
67	23	14.5	6.5	2.5	8
68	24.5	16	7.5	3.5	8
69	36	17	6	3.6	8
70	22.5	11	7.8	2.3	8
71	23.5	12	5	2.3	8
72	23.5	12	5	2.3	8
73	23.5	12	5	2.3	8
74	22	10	6.5	3.5	8
75	23.5	10	6.5	3.5	8
76	36	17	6	3.6	8
77	23.5	10	6.5	2.5	8
78	25	16	8	2.5	8
79	36	17	6	3.6	8
80	24	13	6.6	2.1	8
81	23.5	11	8.5	2.3	8
